# Serologic evidence of human orthopoxvirus infections in Sierra Leone

**DOI:** 10.1186/1756-0500-4-465

**Published:** 2011-10-28

**Authors:** Adam MacNeil, Jason Abel, Mary G Reynolds, RR Lash, Richard Fonnie, Lansana D Kanneh, Willie Robert, Victor K Lungay, Augustine Goba, Lina M Moses, Inger K Damon, Kevin Karem, Daniel G Bausch

**Affiliations:** 1 Centers for Diseases Control and Prevention, Atlanta, GA, USA; 2 Kenema Government Hospital, Ministry of Health and Sanitation, Kenema, Sierra Leone; 3 Tulane School of Public Health and Tropical Medicine, New Orleans, LA, USA

## Abstract

**Background:**

Orthopoxviruses, including variola virus, vaccinia virus, and monkeypox virus, have previously been documented in humans in West Africa, however, no cases of human orthopoxvirus infection have been reported in the region since 1986. We conducted a serosurvey to determine whether human exposure to orthopoxviruses continues to occur in eastern Sierra Leone.

**Findings:**

To examine evidence of exposure to orthopoxviruses in the Kenema District of Sierra Leone, we collected and tested sera from 1596 persons by IgG ELISA and a subset of 313 by IgM capture ELISA. Eleven persons born after the cessation of smallpox vaccination had high orthopoxvirus-specific IgG values, and an additional 6 persons had positive IgM responses. No geographic clustering was noted.

**Conclusions:**

These data suggest that orthopoxviruses continue to circulate in Sierra Leone. Studies aimed at obtaining orthopoxvirus isolates and/or genetic sequences from rodents and symptomatic humans in the area are indicated.

## Background

Orthopoxviruses are large DNA viruses in the family *Poxviridae *[1]. Included in the *Orthopoxvirus *genus are variola virus, the causative agent of smallpox; vaccinia virus, the live virus component of the smallpox vaccine; monkeypox virus, the causative agent of monkeypox; and as well as other zoonotic viruses, including many not known to cause human disease. Smallpox vaccination results in broadly reactive anti-orthopoxvirus serologic responses that persist for decades [2].

**Figure 1:**
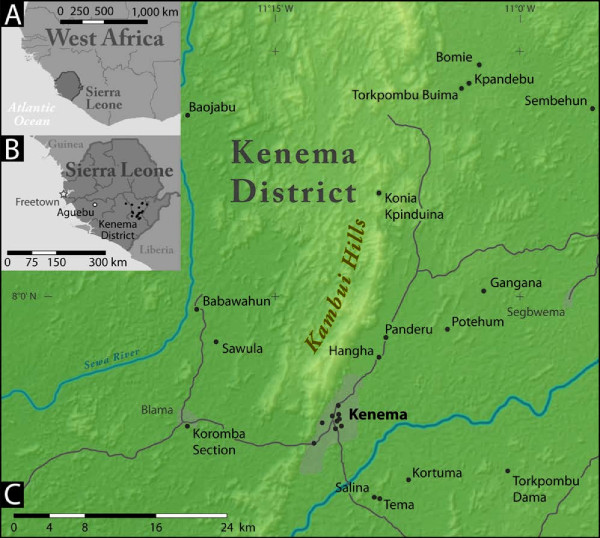
**Location of Sierra Leone in West Africa (A), location of Kenema district in Sierra Leone (B), and location of study villages in Kenema district (C)**. In figure 1B, the star denotes the capitol (Freetown) and the open circle denotes the location of a confirmed monkeypox case in 1970.

Sierra Leone is a country of 6 million inhabitants on the west coast of Africa (Figure 1). In response to an outbreak of smallpox in Sierra Leone in 1967-1968, a large vaccination campaign was launched, resulting in vaccination coverage close to 80% [3]. Although we were unable to verify the precise date that routine smallpox vaccination ceased in Sierra Leone, veteran colleagues in the region put it at 1972-73. Smallpox was declared eradicated in 1980. Numerous cases of human monkeypox were detected in West Africa between 1970 and 1986, including a single case in Sierra Leone in 1970, but no cases have been reported since [4-7]. However, a monkeypox outbreak resulting in 47 confirmed and probable cases occurred in the United States in 2003, and was linked to a West African strain of the virus imported through a shipment of African mammals from Ghana [8-10].

Despite the absence of reported human orthopoxvirus infections in West Africa in recent decades, the introduction of monkeypox virus into the United States from West Africa demonstrated that orthopoxviruses are still present in the region, at least in small mammals. We conducted a large serosurvey in Kenema District in eastern Sierra Leone, to determine whether human exposure to orthopoxviruses continues to occur in that region. This is the most populous district in the country, with an estimated 482,000 inhabitants [11]. Extensive human migration took place in this area of the country during the civil wars in Sierra Leone (1991-2002) and Liberia (1999-2003); nearly 16,000 displaced Sierra Leoneans and Liberians have resettled in Kenema District [12]. Kenema Town, the largest city in the district, is the site of an established government hospital and reference center for the region [13].

## Methods

### Serosurvey design and sample collection

The study was approved by the institutional review boards of CDC and Tulane University Health Sciences Center and the Ethics Committee of the Ministry of Health of Sierra Leone. Sera were collected as part of a study on Lassa fever in Kenema District in the Eastern Province of Sierra Leone.

Statistics Sierra Leone, the main body responsible for government statistics in Sierra Leone, administratively divides the country into "enumeration areas." For the survey, 40 enumeration areas identified through a 2004 national census were selected with probability proportional to population size [11]. Eighteen enumeration areas were visited in June and July 2007. Upon arrival to an enumeration area, study personnel randomly selected a direction and sequentially enrolled the first 10 available households in that direction. A household was defined as a group of persons sharing prepared meals. After obtaining verbal informed consent, demographic information and blood were collected from members of the household over 7 years old. Blood samples were stored in cold boxes for the remainder of the day and then the serum was decanted and stored in a solar-powered refrigerator at 8°C until return to the laboratory in Kenema Town that evening. Because the original design of the study was for Lassa fever, participants were not asked about smallpox vaccination, history of illness with rash, or any other questions specifically pertaining to orthopoxviruses.

### Orthopoxvirus-specific IgG and IgM serology

All samples were tested by ELISA for anti-orthopoxvirus IgG antibodies and a subset for IgM antibodies, as previously described [14,15]. Briefly, antigen for the assays was derived from vaccinia virus from the DryVax vaccine strain passaged 4 times in BSC-40 cells. The IgG ELISA is an indirect assay using purified vaccinia virus coated plates (100 μl at 1.2*10^5 ^PFU/well), a goat anti-human IgG horseradish peroxidase conjugate (1:2000 dilution), and developed with tetramethybenzidine one-component substrate; optical density was read at 450 nm. The IgM ELISA is an indirect capture assay using goat anti-human IgM (1:800 dilution) coated plates, purified vaccinia virus (6.2*10^5 ^PFU/well), anti-variola virus hyperimmune mouse polyclonal ascitic fluid (1:250 dilution), goat anti-mouse IgG horseradish peroxidase conjugate (1:6000 dilution), and developed with tetramethybenzidine one-component substrate; optical density was read at 450 nm. Sera were run in parallel using a quality-controlled lot of reagents on the same day at dilutions of 1:100 for the IgG assay and 1:50 for the IgM assay. Known anti-orthopoxvirus positive and negative human sera were used for assay standardization and as controls. Assay cut off values (COV) were defined as the mean plus three standard deviations of 5 known negative specimens. Results are reported as the optical density minus the COV (OD-COV). Based on previous studies, an equivocal range for IgM results was established as OD-COV of 0.0 to 0.04, with values above 0.04 considered positive [14,15]. Although both assays are specific for the genus *Orthopoxvirus*, they cannot differentiate between antibodies elicited by different orthopoxvirus species or from antibodies induced by previous vaccination.

## Results

**Figure 2:**
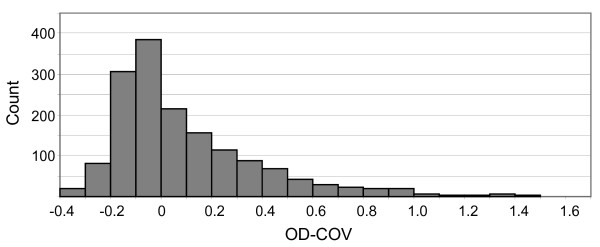
**Distribution of IgG OD-COVs of all study participants (n = 1596)**.

**Figure 3:**
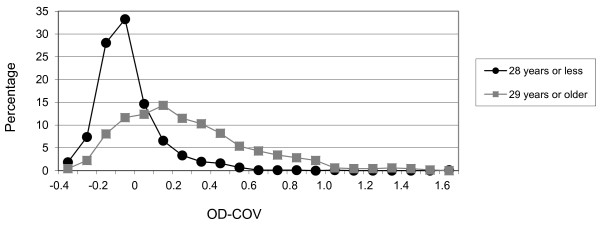
**Distribution of IgG OD-COVs of persons born before (29 years or older, n = 670) and after (28 years or younger, n = 866) the eradication of smallpox in Sierra Leone**. Values on the y-axis represent the percentage of persons in the age group with IgG OD-COVs falling within the plotted range.

Serum samples were obtained from 1596 persons residing in 18 different villages in eastern Sierra Leone (Figure 1). The median age of all participants was 25 years (range 7 to 100 years) and 612 (38.3%) were male. The distribution of IgG OD-COVs fit a pattern similar to a normal distribution, with the highest frequency of values falling between -0.1 and 0, and a modest right skewing, with a low proportion of outlying high values (Figure 2). Because a sizable proportion of the study participants presumably had prior orthopoxvirus exposure due to smallpox vaccination (or possibly due to smallpox), we stratified the sample into those born before (29 years or older, n = 670) and after (28 years or younger, n = 866) the cessation of smallpox vaccination in Sierra Leone, using the year before declaration of eradication (1979) as the last year of possible vaccination. Sixty persons were excluded because their age was not available. The distribution of IgG OD-COVs for the younger age group displayed a steep peak between -0.1 and 0 and fell off sharply in the positive value range, whereas values for the older age group were shifted to the right, with a broad peak between 0.1 and 0.2 (Figure 3). As expected, the median IgG OD-COV for the younger age group was significantly lower than for the older age group (-0.07 versus 0.21, respectively, Wilcoxon rank sum p-value < 0.01).

While it is apparent that IgG seroreactivity is higher on aggregate in the older age groups, the distribution plots did not display a clear demarcation for designation of negative and positive seroreactivity. However, 11 persons (1.3%) below vaccination age (3 persons ≤10 years, 6 persons 11-20 years, and 2 persons 21-28 years) showed exceptionally strong positive IgG results (OD-COV > 0.5), suggesting exposure to orthopoxvirus in the absence of smallpox vaccination. Nine (82%) of the seropositive persons were female; however, the proportion of seropositive females and males was not significantly different (p = 0.14; Fisher's exact test), which was not surprising, given the small sample size. There was no apparent geographic clustering of these 11 persons (2 each from the villages of Babawahun, Hangha, Panderu, and Torkpombu Dama and 1 each from Konia Kpinduina, Potehum, and Kenema Town) (Figure 1).

We then selected, from all study participants, the 160 persons with the highest IgG OD-COVs and tested their sera for IgM antibody. A single person (a 70 year old male from the village of Sawula) was positive. Given the indication of possible recent orthopoxvirus exposure at this site, we decided to test all 46 remaining samples from Sawula (n = 46) as well as all persons below vaccination age with an IgG OD-COV > 0.2 (n = 57), and 50 randomly selected persons from the remaining pool of all samples. Five (3%) of these 153 samples were IgM positive (2 from Kenema Town and 1 each from Sawula, Koromba Section and Gangana). Four of the five persons were below vaccination age while the age of the fifth, from the randomly selected group, was unknown. Although 4 of these 5 IgM positive persons had IgG OD-COV values > 0.0, all were < 0.5.

## Discussion

Our data suggest that, although likely infrequent, human exposure to orthopoxviruses continues to occur in eastern Sierra Leone even after the eradication of smallpox. The rarity of exposure is surprising considering the historic occurrence of monkeypox in the region [5,6], and ecologic niche models [16] that might lead one to assume that the orthopoxviruses and their reservoirs are endemic in Sierra Leone. It is possible that herd immunity from historical smallpox vaccination has posed an immunological barrier to other zoonotic orthopoxvirus infections in humans. If so, infections and disease may increase in the future, as population immunity wanes [17].

Despite the low seroprevalence, the finding of high anti-orthopoxvirus IgG titers in 11 persons below vaccination age and positive IgM titers in 6 persons, 4 of whom were below vaccination age, suggest that human exposure to orthopoxviruses in Sierra Leone does occur. Although we do not have a detailed migration history for each study participant, 15 (88%) of the 17 heads of households of these sero-positive persons reported living in the same village for their entire lives, with the exception of some displacement during the civil war. This observation suggests that infection may indeed have occurred in Sierra Leone.

There are multiple possible explanations for the finding of serological evidence of orthopoxvirus infection, but no reported disease, in the study population: First, human illnesses may be occurring but not being detected or reported. The Sierra Leone civil war has severely impeded surveillance and reporting over the past few decades. It is also possible that cases of monkeypox infection continue to occur but, like in Central Africa, are mistaken for chickenpox [18]. No laboratory network or research project exists in West Africa to aid in distinguishing the two. Secondly, orthopoxvirus infection in Sierra Leone may be sub-clinical (mild or asymptomatic) but eliciting cross-reactive antibody responses. Even documented monkeypox infections may be mild or asymptomatic; clinical symptoms during the 2003 monkeypox outbreak in the United States due to a virus imported from West Africa tended to be less severe compared with symptoms typically described in monkeypox cases in Central Africa [9,19-21]. Furthermore, orthopoxvirus-reactive IgM or T-cell responses were noted in a small number of asymptomatic case contacts [15].

Recent serologic evidence from Ghana showed orthopoxvirus infection to be frequent in rodents and humans with rodent exposure despite the absence of any reported human disease in the area [22]. Similarly, human serosurveys in other geographic locations also suggest human exposure to orthopoxviruses, including in vaccine naïve individuals in the Republic of Congo and Brazil [23,24]. In contrast, no serologic reactivity to orthopoxviruses was identified in 52 children in a study conducted in southern India [25].

Presumably the circulating orthopoxvirus in Sierra Leone is not a human pathogen, however due to broad serologic cross-reactivity between orthopoxviruses, we cannot definitively identify the virus (or viruses) associated with these exposures. While other serologic assays (such as plaque-reduction neutralization test) may be more specific for certain viruses, without a presumptive virus species interpretation of other assays may be equally challenging. Taterapoxvirus, which is closely related to variola virus, is not known to cause human infection but has been found in gerbils in West Africa [26].

We recognize various limitations in our study; interpretation of serosurvey results, particularly with regard to setting cut-off values for a positive serologic response, is always challenging and open to debate. Background smallpox vaccination inevitably further clouds the picture when attempting to assess possible exposure to zoonotic orthopoxviruses. Because of these challenges, we took a conservative approach to the interpretation of our data, choosing to focus our analysis on those with exceptionally high IgG OD-COV values rather than simply reporting the overall orthopoxvirus seroprevalence. However, the 11 IgG positive individuals we identified had OD-COV values that were higher than the majority of OD-COV values in the 29 years and older population (a group likely to have a high overall seroprevalence of orthopoxvirus reactive IgG antibodies due to previous vaccination), strongly supporting the notion that this seroreactivity is the result of previous exposure to wild-type zoonotic orthopoxviruses.

In conclusion, despite the low number of positives, the findings from our study, the historic occurrence of monkeypox in the region [5,6], and ecologic niche models [16] suggest that orthopoxviruses still circulate in Sierra Leone. To further explore and document this, we plan to follow-up this study with investigations of orthopoxviruses in the rodent population as well as targeted studies of pustular rash illnesses in humans in the region. Both studies will aim at isolating virus and/or obtaining sequence data as definitive confirmation of orthopoxvirus circulation.

## Competing interests

The authors declare that they have no competing interests.

## Authors' contributions

AM, MR, ID, KK, and DB developed the study and obtained protocol approvals, RF, LK, WR, VL, AG, and LM assisted in design, sampling, and implementation of the serologic survey, JA performed all study assays, AM and AJ performed analysis and interpretation of serosurvey data, RL and LM engaged in collection and analysis of geographic data, AM, MR, ID, KK, and DB drafted the manuscript. All authors read and approved the final manuscript.
